# Avoiding Liver Injury with Papaverine and Ascorbic Acid Due to Infrarenal Cross-Clamping: an Experimental Study

**DOI:** 10.21470/1678-9741-2016-0081

**Published:** 2017

**Authors:** Serhat Huseyin, Orkut Guclu, Volkan Yüksel, Gulen Sezer Alptekin Erkul, Nuray Can, Fatma Nesrin Turan, Suat Canbaz

**Affiliations:** 1 Department of Cardiovascular Surgery of Trakya University School of Medicine, Edirne, Turkey.; 2 Department of Medical Pathology of Trakya University School of Medicine, Edirne, Turkey.; 3 Department of Biostatistics of Trakya University School of Medicine, Edirne, Turkey.

**Keywords:** Reperfusion Injury, Liver Diseases, Papaverine, Ascorbic Acid, Rats, Models, Animal

## Abstract

**Objective:**

Ischemia-reperfusion injury after acute ischemia treatment is a serious condition with high mortality and morbidity. Ischemia-reperfusion injury may result in organ failure particularly in kidney, lung, liver, and heart. In our study, we investigated the effects of papaverine and vitamin C on ischemia-reperfusion injury developed in the rat liver after occlusion-reperfusion of rat aorta.

**Methods:**

32 Sprague-Dawley female rats were randomized into four groups (n=8). Ischemia was induced with infrarenal aortic cross-clamping for 60 minutes; then the clamp was removed and reperfusion was allowed for 120 minutes. While the control group and the ischemia-reperfusion group did not receive any supplementary agent, two other groups received vitamin C and papaverine hydrochloride (papaverine HCL). Liver tissues were evaluated under the light microscope. Histopathological examination was assessed by Suzuki's criteria and results were compared between groups.

**Results:**

In ischemia-reperfusion group, severe congestion, severe cytoplasmic vacuolization, and parenchymal necrosis over 60% (score 4) were observed. In vitamin C group, mild congestion, mild cytoplasmic vacuolization and parenchymal necrosis below 30% (score 2) were found. In papaverine group, moderate congestion, moderate cytoplasmic vacuolization and parenchymal necrosis below 60% (score 3) were observed.

**Conclusion:**

An ischemia of 60 minutes induced on lower extremities causes damaging effects on hepatic tissue. Vitamin C and papaverine are helpful in reducing liver injury after acute ischemia reperfusion and may partially avoid related negative conditions.

**Table:** 

Abbreviations, acronyms & symbols	
I-R	= Ischemia-reperfusion
Papaverine HCL	= Papaverine hydrochloride

## INTRODUCTION

Ischemia develops during aortic surgery due to aortic clamping in lower extremities and the following reperfusion process brings about a systemic inflammatory response that causes endothelial injury and increasing vascular permeability. Non-cardiac pulmonary dysfunction can be indicated in many patients following an elective aorta surgery and is an important cause of postoperative morbidity^[1]^. During reperfusion, many chemical mediators are released into systemic circulation due to washout effect and contribute to distal organ injuries by organ capillary occlusions through possible microembolies^[2-4]^. Those result with increased permeability, platelet aggregation and necrotic areas due to microvascular endothelial injury in major organs^[5]^. This condition caused pulmonary edema and microproteinuria in the kidney. On the other hand, the effects of distal organ ischemia-reperfusion (I-R) injury on liver are examined in a limited number of studies.

Many treatment strategies have been developed to prevent and to reduce I-R injury. As vitamin C (ascorbic acid) has long been known for its antioxidant effect and papaverine has vasodilator effects, we assume that vitamin C and papaverine could reverse the vasoconstrictive effects of the chemical mediators released during systemic inflammatory response and may reduce I-R injury in hepatic tissue^[6,7]^.

The aim of the present study is to perform a histopathological examination of the effects of vitamin C and papaverine on liver tissues following I-R injury in lower extremities.

## METHODS

### Study Design

The study was performed in Experimental Animals Laboratory after the approval by Local Ethic Committee was obtained (Number: 36/2012). Thirty-two female Sprague-Dawley rats, 3.5-4 months old and weighing about 190-250 g, were used. The animals were randomly divided into four groups (n=8). This experimental study was carried out in accordance with Animals Act and associated guidelines. All animals were handled in accordance with the Guide for the Care and Use of Laboratory Animals.

### Preparation of the animals and the surgical techniques

All animals were given ketamin HCl 40 mg/kg (Ketalar^®^ 50 mg/ml vial, Pfizer Drug Company, Istanbul, Turkey) + xylazine hydrochloride 5 mg/kg (Rompun^®^ 23.32 mg/ml, 50 ml vial, Bayer Drug Company, Istanbul, Turkey) after 8 hours fasting, with intramuscular anesthesia into left foot muscle, ensuring spontaneous respiration during the procedure. The animals were placed in a supine position on the table under a radiant heater. After the area of the skin aseptically prepared, all animals were subjected to median laparotomy from right below the xiphoid to 0.5 cm above pubis; intestines were deviated to the right side, then the infrarenal abdominal aorta and liver were explored through a blunt dissection. All animals were given a low dose (100 unites/kg) of heparin (Nevparin^®^ 25000 IU 5 ml flakon, Mustafa Nevzat Drug Company, Istanbul, Turkey). Fluid resuscitation was supplied with 10 ml/kg 0.9% NaCl through the tail vein. Infrarenal abdominal aorta was clamped with an atraumatic microvascular clamp (Novaclip^®^ 12 mm Angle, Plymouth, USA). After clamping, 5 ml warm saline solution was injected into the peritoneal cavity. To prevent abdominal fluid loss, the skin area was approximated with a suture. After 60 minutes of ischemia, clamp was removed and we waited 2 hours for reperfusion. Ischemia was monitored by loss of pulsation in aorta and reperfusion was monitored by the presence of pulsation in aorta. The animals were sacrificed after the procedure, and the livers of all animals were removed and conserved in 10% formalin.

### Experimental Groups

#### Control Group (Group 1) (n=8)

Rats hepatectomised at the end of the targeted period without any other procedure following anesthesia and median laparotomy.

#### Ischemia-Reperfusion Group (Group 2) (n=8)

Rats hepatectomised at the end of the targeted ischemia-reperfusion duration with induced ischemia-reperfusion following a standard surgical procedure.

#### Ischemia-Reperfusion + Vitamin C group (Group 3) (n=8)

Rats with induced ischemia following a standard surgical procedure, started infusion with 50 mg/kg dose of vitamin C (ascorbic acid) (Redoxan^®^ 500 mg/5 ml vial, Bayer Drug Company, Istanbul, Turkey) 15 minutes before declamping and hepatectomised at the end of the reperfusion duration.

#### Ischemia-Reperfusion + Papaverine Group (Group 4) (n=8)

Rats with induced ischemia following a standard surgical procedure, and started infusion with 1.5 mg/kg dose of papaverine HCl (Papaverin HCl^®^ 0.05 g/ 2 ml, Galen Drug Company, Istanbul, Turkey) 15 minutes before declamping, and hepatectomised at the end of the reperfusion duration.

### Histopathological Examination

Liver tissues were fixed separately in 10% buffered neutral formalin, and tissue monitoring was performed. From the samples, paraffin blocks were prepared and 4-5 µm sections were cut and stained with hematoxylin and eosin (H+E). Histopathological examinations were performed under a light microscope. Sinusoidal congestion, hepatocyte cytoplasm vacuolization and parenchymal necrosis were assessed as described by Suzuki et al.^[8]^ and scored on a scale from 0 to 4 (Table 1).

**Table 1:** Suzuki scoring scale.

Score	Congestion	Cytoplasmic vacuolization	Parenchymal necrosis
0	No	No	No
1	Minimal	Minimal	Single-cell necrosis
2	Mild	Mild	< 30%
3	Moderate	Moderate	< 60%
4	Severe	Severe	> 60%

### Statistical Analysis

Statistical evaluations were performed using Statistical Package for the Social Sciences 20 (SPSS Inc, Chicago, IL, USA) and, for multiple comparisons, Kruskal Wallis analysis of variance and Bonferroni corrected Mann-Whitney U test were used. Descriptive statistics were median (min-max) values. Level of significance was accepted as *P*≤0.05; two-way and *P*≤0.008 for Bonferroni correction.

## RESULTS

**Fig. 1:**
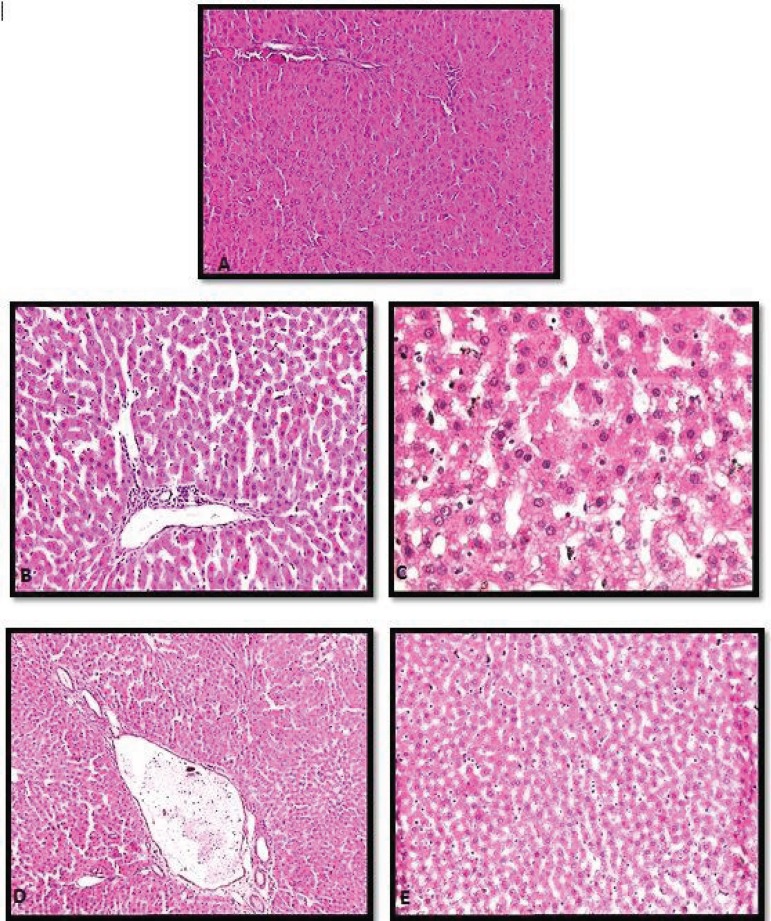
A) Group 1 subject 1, score 0: regular hepatic histology. B) Group 1 subject 8, score 1: sinusoidal congestion findings. C) Group 2 subject 6, score 4: marked congestion, extensive cytoplasmic vacuolization, focal cellular necrosis. D) Group 4 subject 5, score 2: sinusoidal congestion, mild level of cytoplasmic vacuolization. E) Group 4 subject 6. score 3: moderate level of congestion and cytoplasmic vacuolization.

In our study, we used Suzuki classification in assessments and while no pathological differences were observed in the livers of three animals (Suzuki score 0), minimal congestion, minimal cytoplasmic vacuolization, and single-cell necrosis were observed in five animals (Suzuki score 1) in Group 1 (Figures 1A and B).

In Group 2, minimal congestion, minimal cytoplasmic vacuolization, and single-cell necrosis were observed in three rats (Suzuki score 1); mild congestion, mild cytoplasmic vacuolization and parenchymal necrosis below 30% were observed in two rats (Suzuki score 2); moderate congestion, moderate cytoplasmic vacuolization, and parenchymal necrosis below 60% were observed in two rats (Suzuki score 3); severe congestion, severe cytoplasmic vacuolization, and parenchymal necrosis above 60% were observed in one rat (Suzuki score 4) (Figure 1C).

In Group 3, while no histopathological difference in the liver (Suzuki score 0) was observed in one animal, there were minimal congestion, minimal cytoplasmic vacuolization, and single-cell necrosis (Score 1) in five animals, and mild congestion, mild cytoplasmic vacuolization and parenchymal necrosis below 30% (score 2) in two animals (Figure 1D).

In Group 4, although the histopathological examination of the liver revealed no difference (score 0), minimal congestion, minimal cytoplasmic vacuolization, and single-cell necrosis were observed in four animals (Score 1); mild congestion, mild cytoplasmic vacuolization, and parenchymal necrosis below 30% were observed in two animals (Score 2); and moderate congestion, moderate cytoplasmic vacuolization and parenchymal necrosis below 60% were observed (Score 3) in one animal (Figure 1E).

**Fig. 2:**
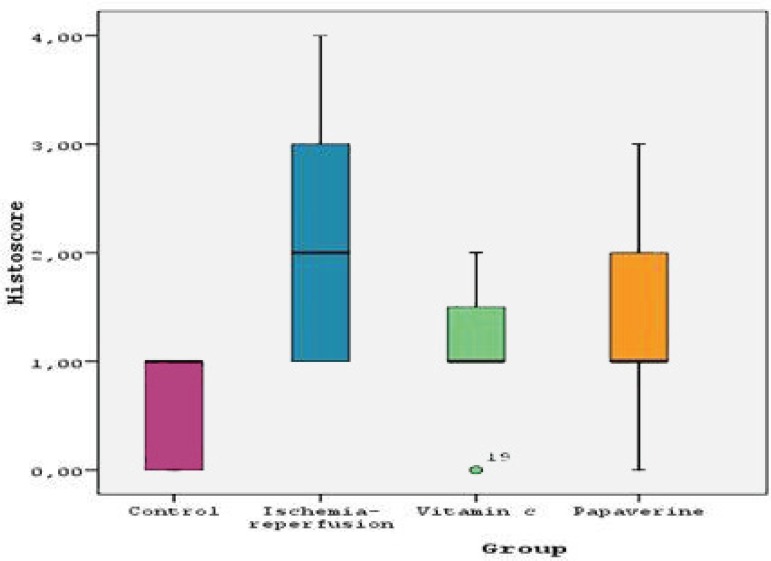
Histopathological scores by groups.

There was a statistically significant difference among the groups Suzuki scores (*P*=0.021). In pairwise comparisons, while a significant difference (*P*=0.007) between Group 1 and Group 2 was found, the comparisons among other groups did not reveal any significant difference (respectively; Group 1 with Group 3, and with Group 4; *P*=0.195; 0.105; Group 2 with Groups 3 and 4; *P*=0.083, 0.234; with Group 2 and Group 3; *P*=0.645). In other words, although similar histopathological values were obtained in the comparison of Groups 3 and 4 with Group 1 and of Group 3 with Group 4, the histopathological scores were lower in Group 1 in comparison to Group 2 (*P*=0.007) (Table 2, Figure 2).

**Table 2:** Histopathological scores by groups.

Group	Median	Minimum	Maximum
Control (n=8)	10.000	0	1.00
Ischemia-reperfusion (n=8)	20.000	1.00	4.00
I-R +Vit C (n=8)	10.000	0	2.00
I-R+ Papaverine (n=8)	10.000	0	3.00

## DISCUSSION

Infrarenal abdominal aortic surgery for abdominal aortic aneurysm and peripheric bypass surgery have remained the gold standard treatment despite the decreasing number of such procedure with the development of endovascular techniques^[9]^. I-R experiments constitute the experimental models simulating injuries developed following such organ surgeries, circulation shocks, and disseminated intravascular coagulation disorders^[10]^. Aortic clamping procedure used in abdominal aortic surgery is not an innocent procedure, as it is well known. Not only its local effects, but also the systemic effects contribute to this fact. In patients with peripheral arterial disease, who have abnormal microvascular perfusion, oxygen increase during reperfusion is higher and therefore, ischemic injury is more severe^[10]^. For this reason, antioxidant agents to reduce the injury are being tested in experimental models which otherwise require a long processes to clinical application^[10]^.

In this study, we used ascorbic acid, because it is the strongest antioxidant soluble in water. We also used papaverine, an opium alkaloid and a good vasodilator that is thought to have an antioxidant effect through inhibition of mitochondrial glutamate oxidase system for decreasing oxidative stress and lipid peroxidation^[10,11-13]^. We found that ascorbic acid and papaverine caused some decrease in I-R related histopathological injury, although not reaching a statistically significant level. Various studies have addressed the use of similar agents in similar durations and their beneficial effects. In experimental studies on rats, reperfusion of distal organ ischemia has been shown to cause injury in lungs, kidneys, and in small intestine, and the use of certain molecules, such as melatonin, oxytocin, ascorbic acid and complement inhibitors, has reductive effects on the injuries^[14-22]^. Strategies directed at reducing apoptosis are thought to improve survival after ischemia and reduce reperfusion injury^[8,23]^.

We need to look at the pathophysiological process, caused by the condition in lower limbs. Skeletal muscle of the lower limbs is the major tissue that is susceptible to ischemia. Irreversible muscle injury starts after 3 hours of ischemia and complete at the 6^th^ hour^[24]^. Mortality and amputation rates in acute peripheric arterial occlusion have been reported as 10-25% and 20%, respectively. Duration of ischemia has great importance among the factors that affect mortality and morbidity^[25]^. Free oxygen radicals, TNF-alpha and other mediators released into circulation during I-R activate various proteins involved in apoptosis. This causes DNA damage and cell death^[14,15,23]^. The more affected tissue mass and duration of ischemia, the more severe are the complications^[24]^.

The effects of I-R injury occur after 45 minutes in lungs and after 60 minutes of ischemia in extremities during reperfusion^[15,26,27]^. The tolerable duration of normothermic hepatic ischemia is not known and thought that irreversible injury occurs after 90 minutes of elective liver surgery^[18]^. However, there is a limited number of clinical and experimental studies on distal organ I-R-related liver injury. In literature, there two studies that have reported the histopathological and biochemical effects of distal organ ischemia reperfusion on liver^[17]^. Based on similar studies, we planned to use I-R duration as 60 minutes of ischemia and 120 minutes of reperfusion^[14,16]^. We also demonstrated in our study that 60 minutes of ischemia and 120 minutes of reperfusion induced in lower limbs of healthy subjects caused a significant I-R injury in hepatic tissue histopathologically.

However, clinical studies have shown that the liver injury occurred in this part is influenced by many other factors, from hepatic lipid content to various systemic factors like acute hyperglycemia^[8,23]^. Sprung et al.^[28]^, in their clinical study on 942 consecutive patients who had elective abdominal aortic aneurysm and vasculopathy, found an acute increase in hepatic enzymes in 1.5% of the patients at 24-72 hours postoperatively. However, we did not analyze the effects of the systemic factors on our subjects due to limited technical resources. This is a limitation in our study. Another limitation is that the subjects in our study do not have any vasculopathy and systemic risk factors, unlike real patient population.

Initiation time of the antioxidant agents is also important. Since the free radicals formation has shown to occur rapidly (in the first seconds of the reperfusion), it has been known that the use of free radical scavengers is only effective when it is initiated 15 minutes prior to reperfusion if it is to reduce the reperfusion injury, and it has not any protective effect when initiated after reperfusion^[2,29]^. In our study, distal ischemia was also induced and at 45 minutes of ischemia, that is, 15 minutes before reperfusion antioxidant agent was initiated and the histopathological results were analyzed by using Suzuki classification^[16]^. Although similar histopathological values were obtained in the comparison of Group 3 and Group 4 with Group 1 and Group 3 with Group 4, the histopathological scores were lower in the control group in comparison with Group 2. In other words, Group 4 and Group 3 revealed similar results, yet the level of injury in these groups were lower compared to Group 2, although not statistically significant. In addition, Andrews et al.^[30]^ demonstrated that, in experimental ischemia model in the gastric mucosa before reperfusion, the use of agents that increase gastric blood flow and vasodilator papaverine do not have any beneficial effect on I-R injury. These findings suggest that papaverine may have different effects depending on the type of ischemia, or on the organ involved. In another study, Koçarslan et al.^[31]^ showed that intraperitoneal administration of silymarin reduces oxidative stress and protects liver, kidney, and lungs from acute supraceliac abdominal aorta ischemia-reperfusion injury in the rat model. Pharmacological, genetic and surgical studies directed at decreasing I-R injury would elucidate the prospect to decrease mortality and morbidity caused by vascular diseases and surgeries.

## CONCLUSION

In conclusion, we revealed that an ischemia of 60 minutes induced on lower limbs is sufficient to cause damaging effects on hepatic tissue. We conclude that both papaverine and ascorbic acid, given 15 minutes prior to reperfusion, reduced hepatic injury caused by reperfusion following ischemia in lower limbs in experimental conditions.

**Table:** 

Authors' roles & responsibilities	
SH	Conception and design study; conduct of procedures and/or experiments; writing of the manuscript or review of its content; final manuscript approval
OG	Analysis and/or data interpretation; final manuscript approval
VY	Manuscript redaction or critical review of its content; final manuscript approval
GSAE	Analysis and/or data interpretation; final manuscript approval
NC	Pathological examination; final manuscript approval
FNT	Statistical analysis; final manuscript approval
SC	Critical review of its content; final manuscript approval
